# Exogenous Melatonin Activates Antioxidant Systems to Increase the Ability of Rice Seeds to Germinate under High Temperature Conditions

**DOI:** 10.3390/plants11070886

**Published:** 2022-03-25

**Authors:** Yufeng Yu, Liyuan Deng, Lu Zhou, Guanghui Chen, Yue Wang

**Affiliations:** 1 College of Agronomy, Hunan Agricultural University, Changsha 410128, China; yyfeng@stu.hunau.edu.cn (Y.Y.); dengliyuan@stu.hunau.edu.cn (L.D.); zhoulu@stu.hunau.edu.cn (L.Z.); 2 The Key Laboratory of Crop Germplasm Innovation and Resource Utilization of Hunan Province, Hunan Agricultural University, Changsha 410128, China

**Keywords:** rice, high temperature stress, melatonin, seed germination, physiology and biochemistry

## Abstract

High temperatures are a major concern that limit rice germination and plant growth. Although previous studies found that melatonin can promote seed germination, the physiological regulation mechanism by which exogenous melatonin mediates high temperature tolerance during rice seed germination is still largely unknown. In order to overcome these challenges, the present study investigates the effects of melatonin on the characteristics of rice seed germination as well as on antioxidant properties, under different high temperature conditions. The results show that 100 μM melatonin seed-soaking treatment under high temperature conditions effectively improves the germination potential, the germination index, and the vigor index of rice seeds; increases the length of the shoot and the root; improves the activity of the antioxidant enzymes; and significantly reduces the malondialdehyde content. The gray relational grade of the shoot peroxidase activity and the melatonin soaking treatment was the highest, which was used to evaluate the effect of melatonin on the heat tolerance of rice. The subordinate function method was used to comprehensively evaluate the tolerance, and the results show that the critical concentration of melatonin is 100 μM, and the critical interactive treatment is the germination at 38 °C and followed by the recovery at 26 °C for 1 day + 100 μM. In conclusion, 100 μM of melatonin concentration improved the heat resistance of rice seeds by enhancing the activity of the antioxidant enzymes.

## 1. Introduction

Rice (*Oryza sativa* L.) is the main food crop for more than 50% of the world’s population [1]. As the global temperature continues to rise, temperature is increasingly becoming a major factor affecting plant growth. In recent years, most of the rice planting areas have suffered from high temperatures and heat damage [2]. Encountering high temperatures above 35 °C during seed germination can reduce seed vigor and hinder germination. Seed germination is the most important developmental stage in the rice life cycle [3]. Temperature is a key factor that affects seed germination. Liu et al. [4] showed that a high temperature treatment of 38 °C for 1–2 days promoted the rapid germination of rice seeds, but the length of the shoot and the root was significantly reduced by 73.7% and 80.8%, respectively. Mangrauthia et al. [5] found that high temperature stress at 42 °C for 5 days inhibited seed growth, reduced the number of seed roots, and seriously affected the protein metabolism process of the rice. In general, excessively high temperatures inhibit seed germination and seedling establishment.

Rice is constantly challenged by various biological and abiotic stresses during its growth and development stages. In order to resist adverse environmental conditions, rice has developed a variety of complex regulatory strategies, including enzymatic and non-enzymatic systems. The enzyme system consists of a series of antioxidant enzymes, such as superoxide dismutase (SOD), peroxidase (POD), catalase (CAT), ascorbic acid peroxidase (APX), and glutathione reductase (GR), which play a major role in scavenging reactive oxygen species (ROS). High temperature has become one of the most important environmental stress factors affecting global agricultural productivity. It is always necessary to cultivate new rice varieties with high temperature tolerance. However, this development is time-consuming and complex. In addition, the exogenous application of some plant growth regulators, such as plant hormones, can effectively overcome the harmful effects of high temperature on plants.

Melatonin (N-acetyl-5-methoxytryptamine) is an indole hormone that is ubiquitous in plants [6]. Melatonin has functions similar to auxin (IAA), which can effectively scavenge free radicals in plants, promote plant growth and development, and improve plant resistance to abiotic and biotic stresses [7]. Another role of melatonin is to protect plants from stressful conditions and to alleviate damage caused by oxidative stress levels at the cellular level [8]. In recent years, many important functions of melatonin have been demonstrated in plants. Melatonin has been postulated to act as a universal antioxidant, circadian cycle regulator, and vegetative growth promoter, which can delay leaf senescence and promote fruit ripening. The exogenous melatonin pretreatment of rice seedlings under cold stress could reduce the accumulation of ROS, the content of malondialdehyde, and cell death as well as relieve the inhibition of stress on photosynthesis and photosystem II activities to improve the activity of antioxidant enzymes and non-enzymatic antioxidant levels to resolve the inhibitory effect [9]. Melatonin pretreatment under salt stress could enhance osmotic regulating substances and adjust ion homeostasis under salt stress to effectively improve the repression of cotton seed germination [10]. The germination indices of wheat variety Hengguan35 with polyethylene glycol (PEG) plus 100 μΜ melatonin and 300 μM melatonin treatments were 29.8–34.5% higher, respectively, compared to PEG alone [11]. Under high temperature stress, exogenous melatonin treatment effectively reduced the oxidative damage of wheat seedlings at high temperatures, decreased the MDA content by almost 5 times at a high temperature of 42 °C at 6 h, delayed leaf aging, and improved the stress tolerance of the plants [12]. Some authors confirmed that melatonin treatment (100 μM), when applied to increase the endogenous melatonin levels and the photosynthetic pigment content along with upregulating their biosynthesis gene expression under high temperature stress (42 °C for 24 h), decreased the values of gas exchange parameters in tomato seedlings, which can mediate photosynthesis performance [13]. Melatonin alleviated the production of ROS in tomato anthers under high temperatures by upregulating the transcription and activity of antioxidant enzymes to effectively improve induced pollen inactivation and inhibit pollen germination [14]. Melatonin could positively alter tea growth and quality by modulating the photosynthesis and the biosynthesis of polyphenols, amino acids, and caffeine in tea leaves under sub-high temperatures [15]. Pretreatment with melatonin increased plant growth and photosynthetic pigments (e.g., chl a and chl b) and reduced oxidative stress by scavenging hydrogen peroxide and superoxide to hinder the MDA and electrolyte leakage contents in soybean plants [16].

According to the current research results, the administration of exogenous melatonin can alleviate the inhibition of plant growth and development under abiotic stress. However, there have been few reports on the alleviating effects of exogenous melatonin treatment on the germination characteristics of rice seeds under high temperature stress. Therefore, this experiment used the rice variety XZX45 to study the effect of melatonin on the germination and physiological characteristics of rice seeds under different high-temperature-stress conditions, and four melatonin concentrations (0, 20, 100, and 500 μM) were used to screen out the most optimal concentration for rice seed germination and growth to explore its physiological regulation mechanism under high temperature stress. This study provides a foundation for the molecular mechanism involved in the melatonin regulation of rice seed germination.

## 2. Results

### 2.1. Germination Characteristics

**Figure 1:**
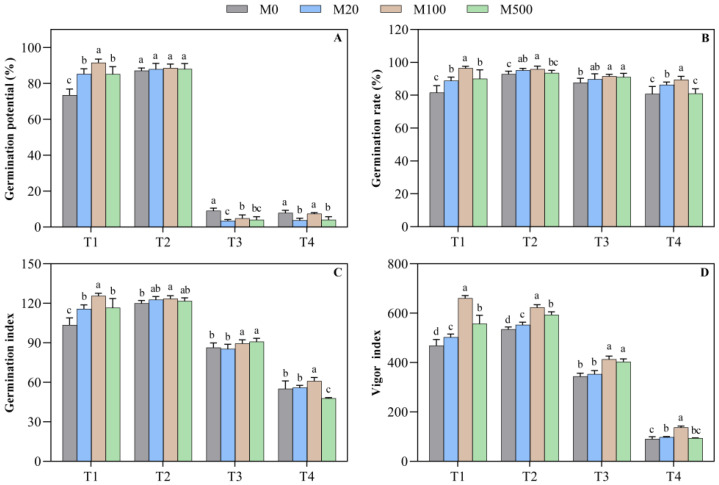
Effects of exogenous melatonin on the germination characteristics of rice seeds under high temperature stress. Means ± SEs with different letters in each parameter indicate significant statistical differences (*p* < 0.05). (**A**) Germination potential; (**B**) germination rate; (**C**) germination index; and (**D**) vigor index.

Compared with M0, the germination potential, the germination rate, the germination index, and the vigor index of each concentration of melatonin in the T1 treatment were significantly increased by 15.9–24.5%, 8.9–18.2%, 11.9–21.4%, and 7.2–40.9%, respectively (Figure 1A–D). In the high-temperature-stress T2 treatment, the vigor index after the melatonin-soaking treatment was significantly increased by 10.9–33.3%, when compared to M0. In the high-temperature-stress T3 treatment, when compared to M0, the germination rate, germination index, and vigor index increased significantly by 4.6%, 3.7%, and 20.5%, respectively. In the high-temperature-stress T4 treatment, the germination rate, germination index, and vigor index of the M100 treatment were significantly higher than the other treatments and were significantly improved by 10.6%, 10.3% and 52.2%, respectively, when compared to the M0 treatment.

### 2.2. Biomass of Rice Seed Shoots and Roots

**Figure 2:**
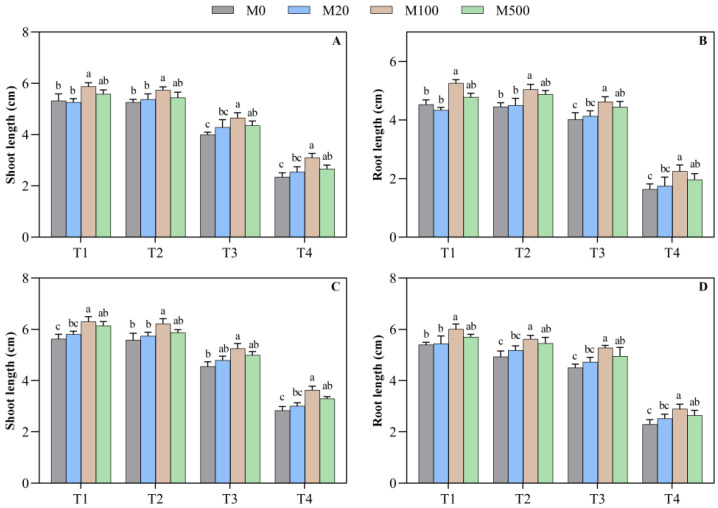
Effects of exogenous melatonin on morphological characteristics of rice seed shoots and roots under high temperature stress. Means ± SEs with different letters in each parameter indicate significant statistical differences (*p* < 0.05). (**A**) Shoot length (7 d); (**B**) root length (7 d); (**C**) shoot length (9 d); and (**D**) root length (9 d).

The seed-soaking treatment with melatonin increased the length of the shoot and the root and was best processed at medium-to-high concentrations (Figure 2A–D). On the 7th day of the high-temperature-stress T2 treatment, when compared to M0, the shoot and root lengths increased by 8.9% and 13.2%, respectively, after the M100 treatment. In the high-temperature-stress T3 and T4 treatments, with the optimal treatment with M100 and M500, when compared to M0, the shoot length increased by 8.8–16.2% and 13.4–32.1%, respectively, and the root length increased by 11.8–16.2% and 19.8–37.6%, respectively. On the 9th day, the shoot and root lengths in the T1 treatment with M100 increased by 11.9% and 11.2%, respectively, when compared to M0. In the high-temperature-stress T4 treatment, M100 and M500 treatments significantly increased the shoot and root lengths by 16.2–27.8% and 15.0–26.1%, respectively, when compared to M0.

**Figure 3:**
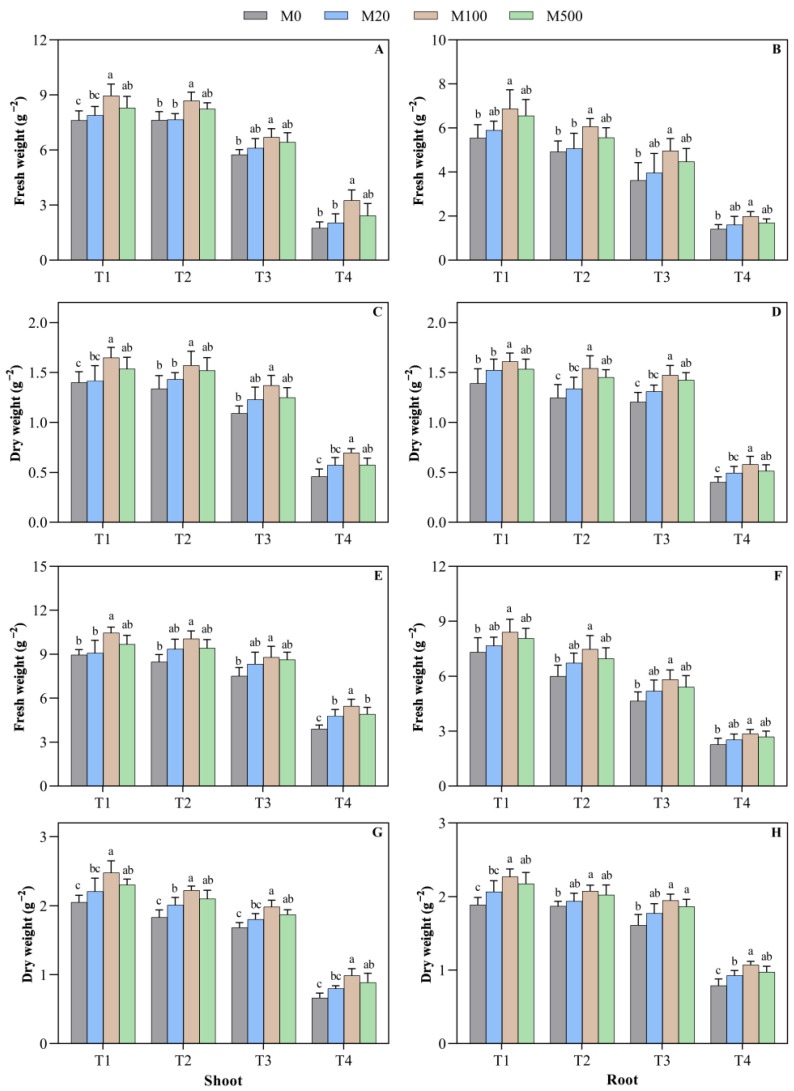
Effects of exogenous melatonin on the rice seed biomass under high temperature stress. Means ± SEs with different letters in each parameter indicate significant statistical differences (*p* < 0.05). (**A**) Shoot fresh weight (7 d); (**B**) root fresh weight (7 d); (**C**) shoot dry weight (7 d); (**D**) root dry weight (7 d); (**E**) shoot fresh weight (9 d); (**F**) root fresh weight (9 d); (**G**) shoot dry weight (9 d); and (**H**) root dry weight (9 d).

Under high temperature stress, melatonin treatment had a promoting effect on the rice seed biomass, and the best results were found with the M100 treatment (Figure 3A–H). On the 7th day of the high-temperature-stress T2, T3, and T4 treatments, when compared to M0, the M100 treatment significantly increased the shoot fresh weight by 13.9%, 16.0%, and 87.4%, respectively, and the shoot dry weight by 23.1%, 37.3%, and 40.1%, respectively. On the 9th day of high temperature stress in T3 and T4 treatments, when compared to M0, the M100 treatment significantly increased the dry weight of the shoot and the root; the shoot dry weight increased by 17.8% and 50.0%, respectively, and the root dry weight increased by 17.8% and 50.0%, respectively.

### 2.3. SOD Activity in Rice Seed Shoots and Roots

**Figure 4:**
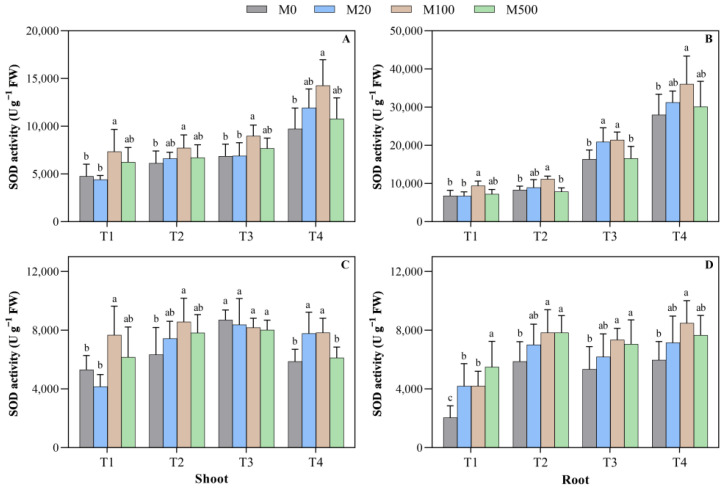
Effects of exogenous melatonin on the superoxide dismutase (SOD) activity of rice seed germination under high temperature stress. Means ± SEs with different letters in each parameter indicate significant statistical differences (*p* < 0.05). (**A**) Superoxide dismutase (SOD) activity of shoot (7 d); (**B**) superoxide dismutase (SOD) activity of root (7 d); (**C**) superoxide dismutase (SOD) activity of shoot (9 d); and (**D**) superoxide dismutase (SOD) activity of root (9 d).

On the 7th day in the T1 treatment, when compared to M0, the SOD activity of the shoot and the root treated with M100 significantly increased by 54.3% and 38.2%, respectively (Figure 4A–D). In high-temperature-stress T2, T3, and T4 treatments, when compared to M0, the M100 significantly increased the diminished SOD activity of the root by 34.0%, 30.8%, and 28.8%, respectively. On the 9th day in the T1 treatment, when compared to M0, the SOD activity of the roots treated with melatonin was 2.0–2.7 times higher. In the high-temperature-stress T2, T3, and T4 treatments, the SOD activity of the root with M100 was higher than that of M0; it had increased by 33.5%, 37.5%, and 42.1%, respectively, a significant difference when compared to the other melatonin treatments.

### 2.4. POD Activity in Rice Seed Shoots and Roots

**Figure 5:**
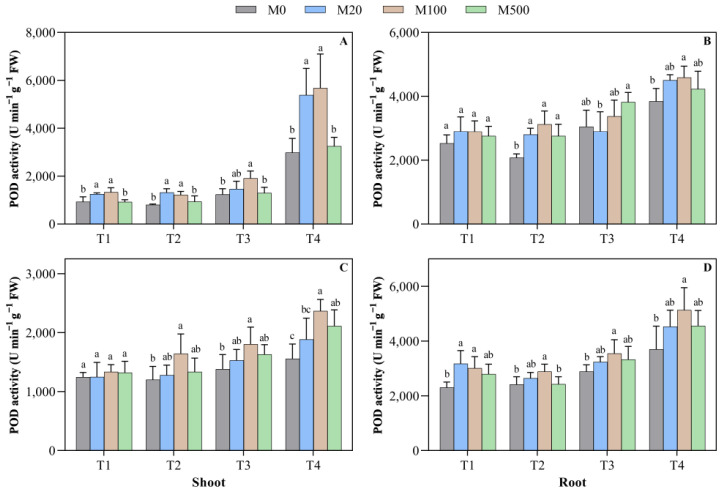
Effects of exogenous melatonin on the peroxidase (POD) activity of rice seed germination under high temperature stress. Means ± SEs with different letters in each parameter indicate significant statistical differences (*p* < 0.05). (**A**) Peroxidase (POD) activity of shoot (7 d); (**B**) peroxidase (POD) activity of root (7 d); (**C**) peroxidase (POD) activity of shoot (9 d); and (**D**) peroxidase (POD) activity of root (9 d).

In the T1 treatment on the 7th day, the POD activity of shoot treated with M20 and M100 significantly increased by 34.9% and 43.8%, respectively, when compared to M0 (Figure 5A–D). In the high-temperature-stress T2 treatment with the melatonin seed-soaking treatment, when compared to M0, the POD activity of the root increased by 34.8–50.6%. In the high-temperature-stress T3 and T4 treatments, when compared to M0, the POD activity of the root treated with M100 significantly increased by 54.2% and 80.7%, respectively. On the 9th day of the high-temperature-stress T2 treatment, when compared to M0, the POD activities of the shoot and the root treated with M100 were significantly increased by 36.7% and 19.5%, respectively. In the high-temperature-stress T4 treatment, when compared to M0, the POD activity of the shoot after melatonin treatment increased by 21.2–54.1%.

### 2.5. CAT Activity in Rice Seed Shoots and Roots

**Figure 6:**
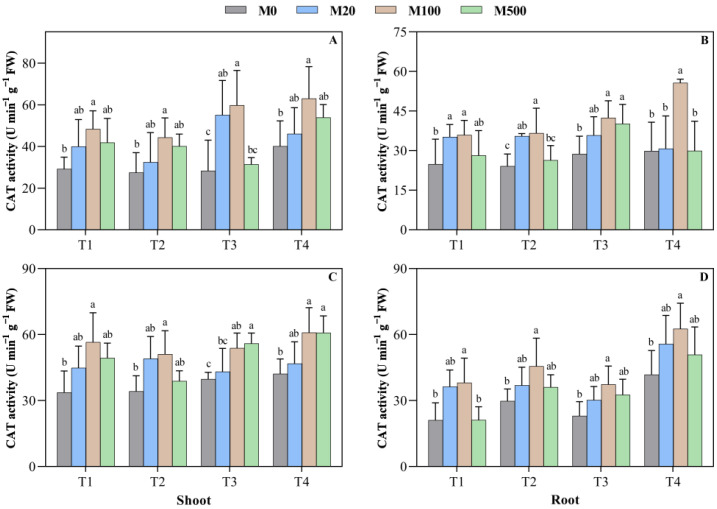
Effects of exogenous melatonin on the catalase (CAT) activity of rice seed germination under high temperature stress. Means ± SEs with different letters in each parameter indicate significant statistical differences (*p* < 0.05). (**A**) Catalase (CAT) activity of shoot (7 d); (**B**) catalase (CAT) activity of root (7 d); (**C**) catalase (CAT) activity of shoot (9 d); and (**D**) catalase (CAT) activity of root (9 d).

On the 7th day of the T1 treatment, when compared to M0, the CAT activity of the shoot and the root treated with M100 was significantly increased by 66.1% and 44.5%, respectively (Figure 6A–D). In the high-temperature-stress T3 treatment, the CAT activity of the shoot with M20 and M100 concentrations were 1.95 times and 2.11 times, respectively, when compared to M0. In the high-temperature-stress T4 treatment, when compared to M0, the CAT activity of the shoot treated with different concentrations of melatonin increased by 15.0–57.4%. On the 9th day of the T1 treatment, the CAT activity of the shoot and the root treated with the M100 concentration of melatonin was 1.68 times and 1.81 times that of M0. In the high-temperature-stress T2 treatment, the M100 concentration of melatonin-treated CAT activity of the root was significantly increased by 53.2%, when compared to M0, and reached a significant level with other treatments. In high-temperature-stress T4 treatment, when compared to M0, M100 and M500 melatonin treatment increased the root CAT activity by 44.4% and 44.3%.

### 2.6. MDA Content in Rice Seed Shoots and Roots

**Figure 7:**
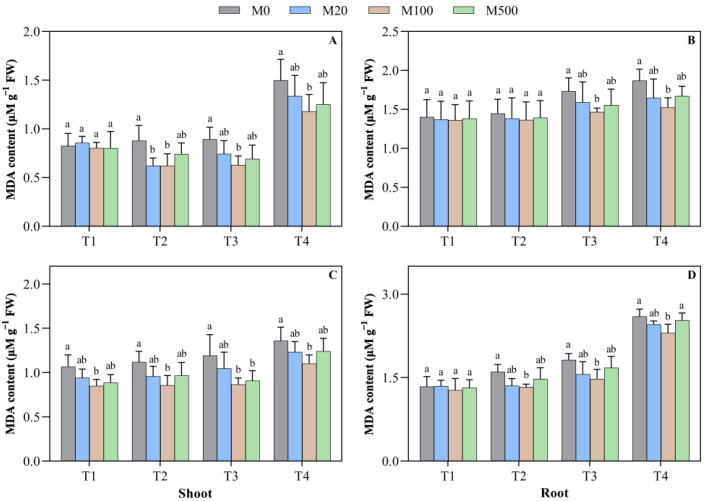
Effects of exogenous melatonin on the malondialdehyde (MDA) content in rice seed germination under high temperature stress. Means ± SEs with different letters in each parameter indicate significant statistical differences (*p* < 0.05). (**A**) Malondialdehyde (MDA) content of the shoot (7 d); (**B**) malondialdehyde (MDA) content of the root (7 d); (**C**) malondialdehyde (MDA) content of the shoot (9 d); and (**D**) malondialdehyde (MDA) content of the root (9 d).

The melatonin seed-soaking treatment significantly reduced the MDA content (Figure 7A–D). On the 7th day of the high-temperature-stress T2 treatment, the MDA content of the shoot treated with melatonin decreased by 15.6–29.3%, when compared to M0. In the high-temperature-stress T3 and T4 treatments, the MDA content of the shoot treated with M100 was significantly higher, when compared to M0, which decreased by 29.5% and 21.2%, respectively. On the 9th day of the high-temperature-stress T2 treatment, when compared to M0, the MDA content of the shoot and the root treated with M100 decreased by 23.3% and 17.2%, respectively. In the high-temperature-stress T3 treatment with M100 and M500, the MDA content of the shoot content was 27.3% and 23.7% lower, respectively, when compared to M0.

### 2.7. Soluble Protein Content in Rice Seed Shoots and Roots

**Figure 8:**
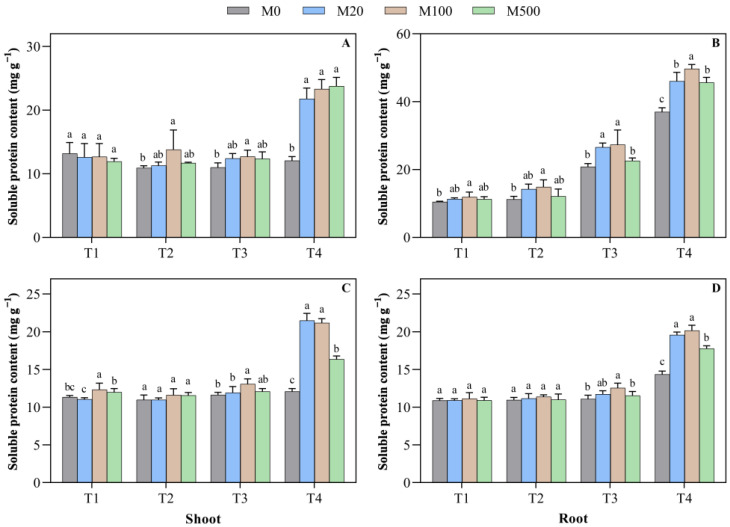
Effects of exogenous melatonin on the soluble protein content in rice seed germination under high temperature stress. Means ± SEs with different letters in each parameter indicate significant statistical differences (*p* < 0.05). (**A**) Soluble protein content of the shoot (7 d); (**B**) soluble protein content of the root (7 d); (**C**) soluble protein content of the shoot (9 d); and (**D**) soluble protein content of the root (9 d).

On the 7th day of the high-temperature-stress T2 treatment, the soluble protein content of the shoot and the root treated with M100 increased by 26.3% and 32.2%, respectively, when compared to M0 (Figure 8A–D). In the high-temperature-stress T4 treatment, the soluble protein content of the shoot and the root with M0 increased by 80.7–97.2% and 23.4–34.3%, respectively. On the 9th day of the high-temperature-stress T3 treatment, the soluble protein content of the shoot and the root treated with M100 increased by 13.4% and 13.1%, respectively, when compared to M0. In the high-temperature-stress T4 treatment, the rice seed soluble protein content of the shoot and the root after the melatonin treatment significantly increased by 35.2–77.6% and 23.9–40.4%, respectively, when compared to M0.

### 2.8. Gray Relational Grade and Correlational Analysis of Exogenous Melatonin in Rice Seed Germination and Physiological Indicators under High Temperature Stress

**Figure 9:**
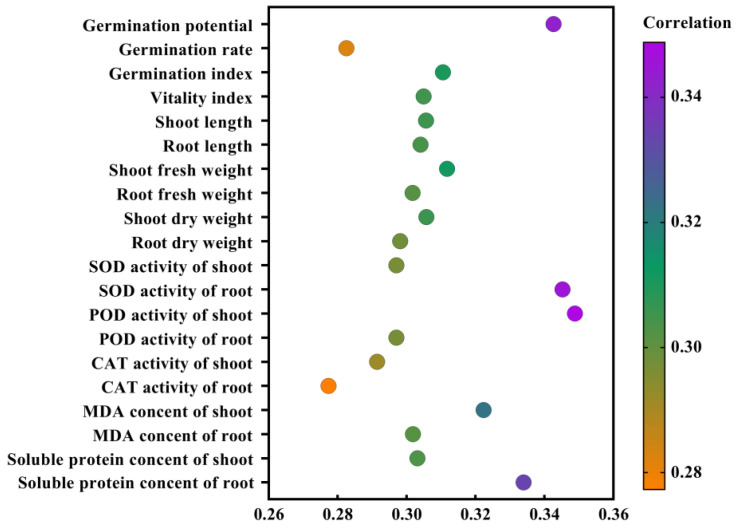
Analysis of the gray relational grade between high temperature stress and rice seed germination and the physiological index under melatonin concentration treatment.

According to the modern agricultural gray system theory, closer changing trends of the experimental array and the reference array indicate a closer mutual relation. The analysis of the gray relational grade between rice seed germination, seedling physiological indices, and melatonin seed-soaking concentrations under high temperature stress is shown in Figure 9. Physiological indicators, such as the POD activity of the shoots, the SOD activity of the roots, and the soluble protein content of the roots, suggested the comprehensive performance of the antioxidant enzyme system in rice seeds, which was closely related to the concentration of melatonin-soaked seeds and could be used as an indicator to measure the effect of melatonin on rice seed germination under high temperature stress. The fresh weight and dry weight of the shoots could also reflect the stress mitigation of the melatonin on rice seeds under high temperatures. Among them, the POD activity of the shoots had the greatest gray relational grade with the concentration of melatonin, which could best reflect the stress mitigation of melatonin.

**Figure 10:**
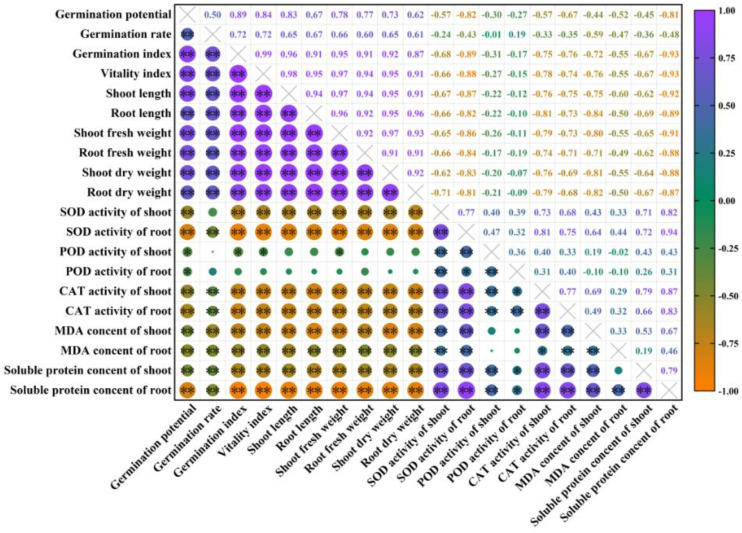
Correlational analysis of seed germination and physiological indices of rice under high temperature stress and melatonin concentration treatment. **: significant at the 0.01 probability level (*p* < 0.01); *: significant at the 0.05 probability level (*p* < 0.05).

The correlational analysis shows that the germination potential and the POD activity have a significant positive correlation (*p* < 0.05), and both the germination and growth indices have a very significantly positive correlation (*p* < 0.01), and the SOD activity, the CAT activity, the MDA, and the soluble protein content have very significantly negative correlations (*p* < 0.01) (Figure 10). The germination rate did not have a significant correlation with the POD activity and the SOD activity of shoot, but had a very significantly positive correlation with the germination and growth indices (*p* < 0.01) and a very significantly negative correlation with the CAT activity, the MDA content, and the soluble protein content (*p* < 0.01). The germination index, the vigor index, the shoot and root lengths, and dry and fresh weights were similar to the germination rate. The SOD activity, the CAT activity, and the soluble protein content were significantly negatively correlated with the germination and growth indices (*p* < 0.01) and significantly positively correlated with the antioxidant enzyme activity and the MDA content (*p* < 0.01).

### 2.9. Principal Component Analysis

**Figure 11:**
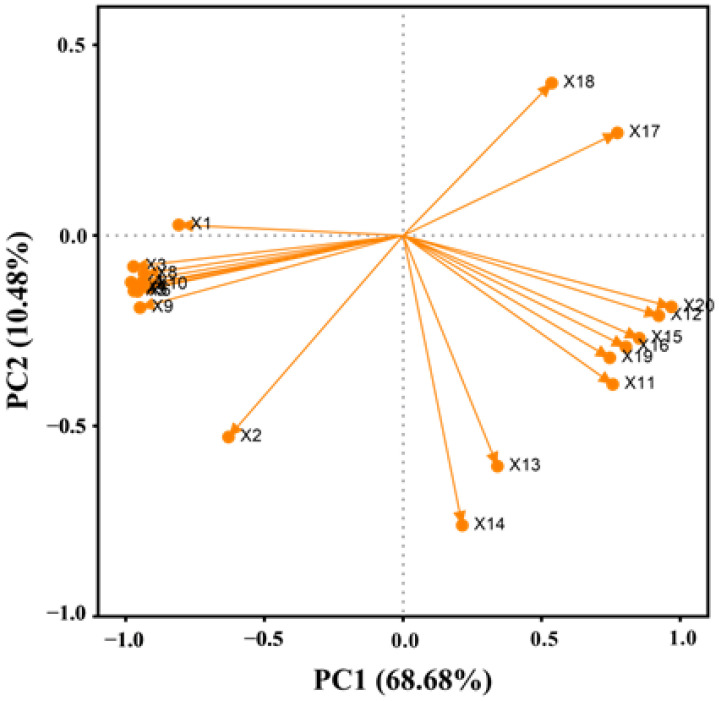
PCA analysis of seed germination and physiological indices of rice under high temperature stress and concentrated melatonin treatment. X1: germination potential; X2: germination rate; X3: germination index; X4: vigor index; X5: shoot length; X6: root length; X7: shoot fresh weight; X8: root fresh weight; X9: shoot dry weight; X10: root dry weight; X11: SOD activity of shoot; X12: SOD activity of root; X13: POD activity of shoot; X14: POD activity of root; X15: CAT activity of shoot; X16: CAT activity of root; X17: MDA content of shoot; X18: MDA content of root; X19: soluble protein content of shoot; and X20: soluble protein content of root.

The principal component analysis (PCA) was used to evaluate the 20 indicators of rice seed germination and physiology under high temperature stress (Figure 11). The results show that the cumulative contribution rate of PCA1 and PCA2 is 79.16%, which reflects the effect of melatonin-soaking on rice seeds under high temperature stress. The characteristic value of PCA1 was 13.74, and the contribution rate was 68.68%. Among them, the indices with large positive characteristic values included SOD activity, CAT activity, MDA content, and soluble protein content. The indices with a large absolute value of negative characteristics included rice seed germination and growth. It showed that when the PCA1 value increased, the value of the positive indicator also increased, while the value of the negative indicator decreased accordingly. The characteristic value of PCA2 was 2.09 and the contribution rate was 10.48%. The indices with large positive eigenvalues included germination potential and MDA content, while other indices were negative eigenvalues, in which the POD activity was highly negatively correlated.

### 2.10. Comparison of the Differences and Comprehensive Evaluation of Tolerance between High Temperature Stress and Exogenous Melatonin Interaction

The results of the two-factor analysis of variance for the effects of high temperature stress and exogenous melatonin concentration on rice seed germination and physiological indices are shown in Table 1. The germination potential, the germination rate, germination index, vigor index, CAT activity of the shoot, soluble protein content of shoot, and soluble protein content of root in rice seeds showed significant differences under high-temperature-stress treatment, exogenic melatonin concentration treatment, and their interaction treatment (*p* < 0.01). Under high-temperature-stress treatment, exogenic melatonin concentration treatment, and their interaction treatment, the significance of shoot length and root length, shoot and root fresh weight, shoot and root dry weight, the SOD activity of shoot and root, the POD activity of the shoot, the CAT activity of the root, and the MDA content of the shoot in rice seeds were different. The difference of each index under single-factor treatment was significant (*p* < 0.01), but there was no significant difference in each index with the interactive treatment. The POD activity of the root of the rice seeds showed a significant difference under a high-temperature-stress treatment (*p* < 0.05) and a significant difference with exogenic melatonin treatment (*p* < 0.01), but there was no significant difference with the interactive treatment. The MDA content of the root of the rice seeds showed a significant difference under high-temperature-stress treatment (*p* < 0.01), but there was no significant difference with the exogenic melatonin treatment or the interactive treatment.

**Figure 12:**
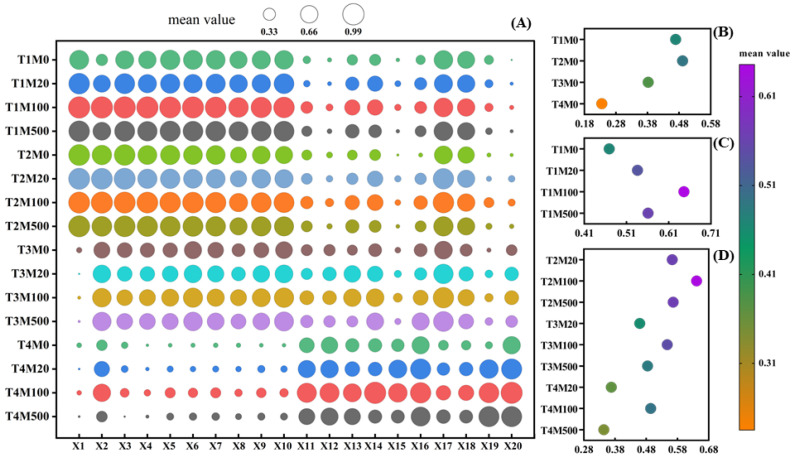
Comprehensive evaluation of rice seed tolerance under the high temperature stress and melatonin single and interactive treatment. (**A**) is the subordinate function value of each index under single and interactive treatment. (**B**–**D**) are the average subordinate function values of single high temperature treatment, single melatonin treatment, and interactive treatment, respectively. X1: germination potential; X2: germination rate; X3: germination index; X4: vigor index; X5: shoot length; X6: root length; X7: shoot fresh weight; X8: root fresh weight; X9: shoot dry weight; X10: root dry weight; X11: SOD activity of shoot; X12: SOD activity of root; X13: POD activity of shoot; X14: POD activity of root; X15: CAT activity of shoot; X16: CAT activity of root; X17: MDA content of shoot; X18: MDA content of root; X19: soluble protein content of shoot; and X20: soluble protein content of root.

In this study, the subordinate function method was used to comprehensively evaluate the tolerance of high temperature stress, melatonin seed-soaking concentration, and the interaction of the two treatments (Figure 12A–D). With a single treatment of melatonin soaking, the mean value of the subordinate function of the T1M100 treatment was the largest, and with the interactive treatment of high temperature stress and melatonin, the mean value of the subordinate function of the T2M100 treatment was the largest.

## 3. Discussion

High temperature stress is one of the most common abiotic stresses in crops and has become a serious environmental concern that affects crop growth. The current research provided insights into the application of melatonin in improving the high temperature tolerance of different crop varieties [17,18]. Rice is one of the most important food crops in the world. Rice seed germination is the most important and complex process in the growth cycle, which can directly affect the development of rice plants and, ultimately, the yields. High temperatures significantly limit the germination and the growth of rice seeds. Melatonin is an environmentally friendly bioactive molecule that plays an important role in mitigating high temperature stress in rice. Previous studies found that soaking seeds with melatonin (100 μM) could have the potential to protect wheat seeds from chromium toxicity. It effectively improved the germination under chromium stress by enhancing the reserve mobilization and antioxidant metabolism of wheat [19]. The results in our experiment indicate that melatonin increases the germination rate of seeds and promotes the subsequent growth in a concentration-dependent manner, but this effect is offset at high concentrations. Soaking seeds with melatonin (100 μM) under 4 different high-temperature conditions (i.e., T1, T2, T3, and T4) significantly reduced the effect of high temperatures on the germination rate by 18.2%, 3.2%, 3.9%, and 10.6%, respectively, when compared to the M0 (0 μM) treatment. In this experiment, for the seeds that were restored to 26 °C normal temperature after a longer duration of the 38 °C high-temperature treatment, the shoot and root lengths, the shoot and root fresh weights, as well as the shoot and root dry weights showed more serious damage to the rice seeds. A total of 100 μM of melatonin treatment could improve these indicators and could effectively alleviate the damage caused by high temperature stress and promote the growth of rice seeds. As was consistent with earlier investigations, drought stress had negative effects on the growth of maize seedlings, such as decreased biomass accumulation. However, the application of melatonin (100 μM) promoted the growth of plants [20].

The application of melatonin to enhance high-temperature tolerance depends on the direct elimination of ROS, the improvement of antioxidant enzyme activity and photosynthetic efficiency, and the regulation of the transcription of the genes related to high temperature stress [13,14]. Melatonin has a dose-dependent effect. The optimal concentration of melatonin has been different in different plant species and plays an important role in resisting environmental stress [19]. In response to the oxidative stress caused by adverse environments, taller plants have formed a set of antioxidant enzyme systems, including SOD, POD, and CAT [21]. In our experiment, soaking rice seeds with melatonin (100 μM) increased the SOD, CAT, and POD activities as well as the soluble protein content of the rice seeds and reduced the MDA content. This indicated that under high temperature stress, melatonin treatment could scavenge ROS produced by regulating the antioxidant enzyme system of rice seeds and reduce the degree of lipid oxidation in rice. Among them, the M100 treatment significantly reduced the toxic effects of oxidative stress and cellular lipid peroxidation in rice under high temperature stress. The antioxidant enzymes in the enzyme system play a special role in the detoxification of ROS. Various protective enzymes in plants coordinate and interact with each other to improve the stress tolerance. SOD is a metalloenzyme that plays a central role in the ROS-scavenging enzyme system. The superoxide anion radical (O^2−^) is disproportioned by SOD to produce H_2_O and O_2_ so as to avoid or reduce the damage of O^2−^ to the cell membrane, which is critical to the dynamic balance of ROS. However, the disproportionation reaction produces H_2_O_2_ intermediary products, damages plant cell membranes, and accelerates plant aging. POD and CAT can further convert H_2_O_2_ into O_2_ and H_2_O through redox, eliminate the toxic effect of H_2_O_2_, and protect plants [22]. In this study, the activities of SOD, POD, and CAT were enhanced in rice seeds that were pretreated with melatonin under high temperature conditions, which also promoted the removal of H_2_O_2_ and improved the antioxidant capacity of the rice seeds. However, the main role of melatonin in the elimination of ROS and whether it is mediated by the enzyme systems during rice seeds germination requires further study. The positive effects of melatonin on the antioxidant enzymes system under high temperature conditions was been confirmed in tall fescue seedlings [21] and *Pinellia ternata* seedlings [23].

In the gray relational grade analysis, many factors (i.e., traits) are regarded as gray systems and integrated for a unified comparison. The importance of each factor is expressed according to the relational grade and order. A large relational grade indicates that the reference index is important. The change of relational grade order means the change of the relationship between factors, which overcomes the subjectivity and the complexities of the previous evaluation of traits. At the same time, when an individual of the sample changes, the value of the relational grade also changes, but the ranking of relational grade remains stable. The relative importance of factors also remains stable, which can then more accurately indicate the impact of each trait on the yield [24]. In this study, the gray relational grade analysis was used to comprehensively evaluate the correlation of melatonin on various trait indicators of rice seedlings. The results show that the antioxidant enzyme activity, the MDA content, and the soluble protein content accurately reflect the stress mitigation of melatonin, particularly under high temperature conditions. This also suggested that under high temperature stress, to further promote the germination and growth of rice seeds, it is necessary to improve the activity of the antioxidant enzymes in plants. Among the indicators, the POD activity of the rice shoot was most closely related to the concentration of melatonin. Therefore, the POD activity of the shoot is the first choice for evaluating the effect of melatonin on the alleviation of high temperature stress, which is the fundamental factor determining the high temperature tolerance of rice seeds. Therefore, in the evaluation of high-temperature-resistant germplasm and variety breeding of rice seeds, we should strengthen the selection and the utilization of the POD activity of the shoot, establish rice seed selection technology based on physiological and biochemical traits, and improve the efficiency and accuracy of the selection. The correlational analysis showed that rice seed germination was significantly positively correlated with the growth of the shoot and the root as well as the dry and fresh weights (*p* < 0.01), and was significantly negatively correlated with the SOD activity, the CAT activity, the MDA content, and the soluble protein content (*p* < 0.01). Through two-way analysis of variance, it was found that a single melatonin treatment and an interactive melatonin treatment could increase the activity of antioxidant enzymes and improve the adaptability of rice in a high temperature environment. In the correlational analysis between the germination rate and the heat resistance index, we found a very significantly negative correlation between the germination rate and the MDA content, indicating that the higher the MDA content, the lower the germination rate, and the more unfavorable for rice seed germination. Previous studies have also shown that high temperature stress can cause membrane lipid peroxidation, damage to membrane structures and functions, and weaken tissue-reducing power, so as to inhibit rice seed germination [16]. The differences in heat tolerance of rice seeds treated with different concentrations of melatonin were evaluated by a comprehensive analysis of subordinate function, and it was found that the M100 concentration provided a stronger tolerance under high temperature treatment, which was consistent with the results of previously measured physiological indicators. To date, this is the first study to explore the mechanism by which melatonin mitigates the inhibitory effects of rice seed germination under high temperature stress. It may provide a foundation for future innovations regarding melatonin modulation in the regulation of plant heat resistance. Moreover, these results provide a theoretical basis for melatonin to alleviate high temperature stress in rice. In the future, we would like to explore the molecular mechanism of melatonin regulation with respect to rice resistance to high temperature stress.

## 4. Materials and Methods

### 4.1. Test Material

The experimental material was XZX45 rice seeds, which were provided by the Rice Research Institute of Hunan Agricultural University. All rice seeds were harvested in July 2020. XZX45 is a rice cultivar that is widely grown in the Yangtze Valley double-crop rice region of China.

Melatonin (N-acetyl-5-methoxytryptamine, MT) was obtained from Sigma-Aldrich (St. Louis, MO, USA). All chemicals were of analytical grade in this study.

### 4.2. Experimental Design

Selected healthy and plump rice seeds of the same size were sterilized with 5% sodium hypochlorite solution for 20 min and then rinsed 4 times with sterilized distilled water. After the seeds were sterilized, they were soaked with different concentrations of melatonin for 24 h, each 100 seeds were sown in repetition, and each treatment had 6 repetitions for the determination of physiological and biochemical indicators. The following melatonin solution treatments were used: M0 (0 μM melatonin, i.e., distilled sterile purified water only), M20 (20 μM melatonin), M100 (100 μM melatonin), and M500 (500 μM melatonin). The seeds were evenly sown in a germination box covered with sterilized germination paper, and an appropriate amount of sterilized distilled water was added, and then they were placed in a light incubator. The light conditions were as follows: fluorescent light with intensity expressed as a PPFD of 150 mmol m^−2^ s^−1^ for 14 h/day, and 70% relative humidity (RLD-1000E-4, Ningbo Ledian Instrument Manufacturing Co., Ltd., Ningbo, China). The high-temperature-stress treatments were as follows: T1 (germinated continuously at 26 °C in the light incubator), T2 (germinated at 38 °C for 1 day and then at 26 °C in the light incubator), T3 (germinated at 38 °C for 2 days and then at 26 °C in the light incubator), and T4 (germinated at 38 °C for 3 days and then at 26 °C in the light incubator). During the germination of rice seeds, the number of germinated seeds was observed and recorded every day. The shoots and the roots of the rice seeds were obtained at 7 d and 9 d, respectively, to determine the relevant indicators.

### 4.3. Measurement Items and Methods

#### 4.3.1. Determination of Relevant Indicators of Seed Germination

The germination of seeds was recorded daily. Seeds were considered to be germinated when the total shoot length exceeded half the length of the seeds and root length exceeded the length of the seeds. The germination potential (GP) and germination rate (GR) were investigated on days 3 and 7 after initiation, respectively. The GP was calculated as GP (%) = (number of seeds germinated on day 3/total number of experimental seeds) × 100. The GR was calculated as GR (%) = (number of seeds germinated on day 7/total number of experimental seeds) × 100. The germination index (GI) was calculated as GI = Σ(Gt/Tt), where Gt = the number of germinated seeds per day corresponding to Tt, and Tt = day of germination test. The vigor index (VI) was calculated as VI = GI × S, where S = root length of germinated seeds on day 7.

#### 4.3.2. Determination of Seed Shoot and Root Growth Indicators

On the 7th day after sowing, 20 plants from each treatment were obtained to measure the growth of the shoot and the root, which were divided into 4 replicates. The measurement of shoot length was determined by the base of the root to the growth point; the measurement of the root length was based on the length of the main root.

#### 4.3.3. Determination of the Biomass Indicators

Consistent with the plant for measuring the growth of the shoot and the root, after measuring the previous indicators, we separated the root and the shoot of the rice, and weighed it with a scale, recorded the fresh weight of the shoot and the root, respectively, and then packed them into bags. They were marked, fixed at 105 °C for 30 min, dried at 65 °C to constant weight, and recorded the dry weight with a balance.

#### 4.3.4. Determination of the Physiological and Biochemical Indicators

The superoxide dismutase (SOD) activity was measured by the nitro blue tetrazolium method [25], the peroxidase (POD) activity was measured by the guaiacol method [26], the catalase (CAT) activity was determined by the UV absorption method [27], the malondialdehyde (MDA) content was determined by the thiobarbituric acid colorimetric method [28], and the soluble protein content was determined by the Coomassie brilliant blue G-250 staining method [29].

### 4.4. Data Processing and Analysis

The data were sorted and calculated using Microsoft Excel 2016 software. The SPSS statistics 20.0 data processing system was used for the statistical analysis, GraphPad Prism 9.3.1 was used for drawing, and Duncan’s new complex difference method was used for the difference test at the level of *p* ≤ 0.05. The gray relational grade analysis referred to the method of Su et al. [30]. The subordinate function method was used to comprehensively evaluate the tolerance of rice seeds to melatonin, high temperature single stress, and interactive stress [31]. The specific subordinate function value calculation formula of each index of each sample was as follows:

Xu = (X − Xmin)/(Xmax − Xmin)(1)

Xu = 1 − (X − Xmin)/(Xmax − Xmin)(2)

In the formula, X is the measured value of a resistance index of the test sample, and Xmax and Xmin are the maximum and minimum values of the index in all samples, respectively. If the measured indices were positively correlated with the tolerance of rice seeds, formula (1) was used to calculate the subordinate function value, and formula (2) was used for the negative correlation. Finally, the subordinate function values of each index of each sample were accumulated, and the average value was obtained.

## 5. Conclusions

**Figure 13:**
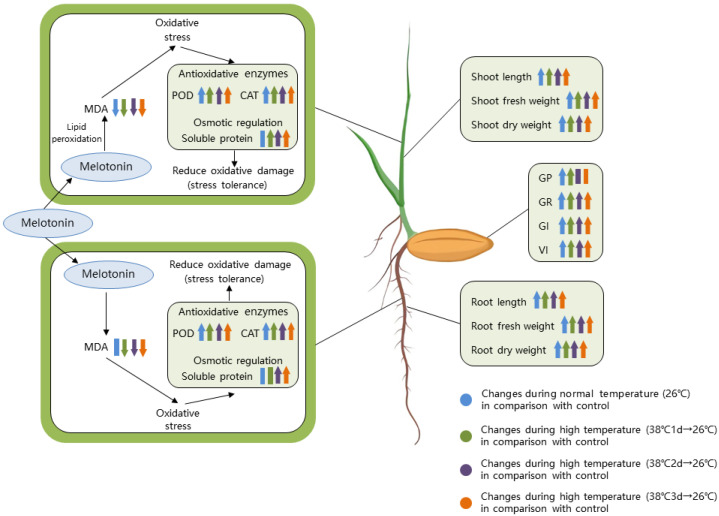
Schematic diagram of the changes in the morphophysiological parameters when 100 μM melatonin alleviates high temperature stress in rice seed germination. (↑), (↓), (|) and other symbols represent up-regulation and down-regulation, respectively, and there is no significant change in each parameter.

In conclusion, melatonin pretreatment can promote rice seed germination and seedling growth, improve agronomic traits, such as shoot length, root length and biomass, increase the antioxidant enzyme system activity and the soluble protein content, and reduce the malondialdehyde content, thereby alleviating the inhibitory effect of high temperature stress on rice growth. The results provide insights into how to utilize melatonin as a novel bioactive molecule to improve heat tolerance in rice. Figure 13 shows the schematic diagram of the physiological and biochemical mechanism involved in alleviating high temperature stress in response to 100 μM melatonin.

## Figures and Tables

**Table 1:** Two-factor analysis of variance for the effects of high temperature stress and exogenous melatonin concentration on rice seed germination and physiological indices.

Index	High Temperature Treatment			MT Treatment			Interactive Treatment		
	Df	F	P	Df	F	P	Df	F	P
X1	3	15,998.1	<0.001	3	19.7	<0.001	9	35.6	<0.001
X2	3	72.3	<0.001	3	42.8	<0.001	9	7.4	<0.001
X3	3	2524.2	<0.001	3	35.0	<0.001	9	15.7	<0.001
X4	3	8352.8	<0.001	3	345.3	<0.001	9	39.3	<0.001
X5	3	846.3	<0.001	3	32.5	<0.001	9	0.8	0.643
X6	3	850.7	<0.001	3	42.1	<0.001	9	1.0	0.45
X7	3	492.9	<0.001	3	19.1	<0.001	9	0.3	0.962
X8	3	192.5	<0.001	3	11.5	<0.001	9	0.4	0.92
X9	3	269.9	<0.001	3	16.0	<0.001	9	0.3	0.964
X10	3	376	<0.001	3	18.1	<0.001	9	0.5	0.89
X11	3	41.8	<0.001	3	8.3	<0.001	9	0.8	0.591
X12	3	169.4	<0.001	3	6.0	0.001	9	0.7	0.743
X13	3	4.6	0.006	3	10.0	<0.001	9	1.3	0.25
X14	3	3.0	0.039	3	11.8	<0.001	9	2.0	0.061
X15	3	146.3	<0.001	3	16.5	<0.001	9	5.3	<0.001
X16	3	56.9	<0.001	3	7.7	<0.001	9	1.8	0.091
X17	3	58.9	<0.001	3	6.0	0.001	9	0.9	0.563
X18	3	8.4	<0.001	3	2.3	0.093	9	0.4	0.947
X19	3	126.8	<0.001	3	22.1	<0.001	9	14.1	<0.001
X20	3	1211.2	<0.001	3	34.9	<0.001	9	6.4	<0.001

Note: X1: germination potential; X2: germination rate; X3: germination index; X4: vigor index; X5: shoot length; X6: root length; X7: shoot fresh weight; X8: root fresh weight; X9: shoot dry weight; X10: root dry weight; X11: SOD activity of shoot; X12: SOD activity of root; X13: POD activity of shoot; X14: POD activity of root; X15: CAT activity of shoot; X16: CAT activity of root; X17: MDA content of shoot; X18: MDA content of root; X19: soluble protein content of shoot; and X20: soluble protein content of root.

## Data Availability

Not applicable.
